# IMAU-Net: A Hybrid Multi-Scale Deep Learning Framework for Liver Segmentation from Laparoscopic Images

**DOI:** 10.3390/s26092695

**Published:** 2026-04-27

**Authors:** Syeda Sitara Waseem, Sarang Shaikh, Syed Rizwan Hassan

**Affiliations:** 1 Department of Computer Science & IT, The Government Sadiq College Women University, Bahawalpur 63100, Pakistan; 2 Department of Information Security and Communication Technology (IIK), Norwegian University of Science and Technology (NTNU), Teknologivegen 22, 2815 Gjøvik, Norway; 3 Department of Computer Engineering, Gachon University, Seongnam-si 13120, Republic of Korea

**Keywords:** liver segmentation, laparoscopic surgery, deep learning, InceptionV3, Multi-Core Pooling, Atrous Spatial Pyramid Pooling, real-time segmentation

## Abstract

Accurate liver segmentation in laparoscopic surgery is critical but remains challenging due to low contrast, occlusion, and irregular organ boundaries. While deep learning has advanced medical image segmentation, existing models often trade off between accuracy, computational efficiency, and boundary precision. We propose IMAU-Net, a hybrid architecture integrating a pre-trained InceptionV3 encoder with a novel bottleneck combining Multi-Core Pooling (MCP) and enhanced Atrous Spatial Pyramid Pooling (ASPP). The MCP module captures fine-to-medium spatial details through parallel multi-kernel pooling, while ASPP extracts multi-scale contextual information via dilated convolutions. Evaluated on the M2CAI dataset with 5-fold cross-validation, IMAU-Net achieves a mean Dice coefficient of 0.9179 ± 0.012 and IoU of 0.8483 ± 0.015. Furthermore, external validation on the independent CholecSeg8K dataset (250 test samples) demonstrates generalizability across different laparoscopic procedures, achieving a Dice coefficient of 0.8745 ± 0.0312 and AUC of 0.9542, with a performance degradation of only 4.3% despite domain shift between liver surgery and cholecystectomy. Comparative analysis with state of the art methods demonstrates superior performance, with computational efficiency suitable for real-time applications (45 FPS, 42.3 M parameters). The proposed architecture provides an optimal balance between accuracy and efficiency for intraoperative guidance systems. While evaluated on retrospective laparoscopic image datasets rather than real-time intraoperative workflows, the model demonstrates potential for integration into surgical guidance systems pending prospective validation.

## 1. Introduction

Laparoscopic liver surgery is more prevalent today because it is minimally invasive, but precise intraoperative segmentation and identification of the liver is still a significant problem. The laparoscopic setting adds complications like low contrast, non-uniform illumination, occlusion by instruments, and irregular boundaries of organs, all of which make pixel-level segmentation procedures difficult [1,2,3]. Traditional solutions cannot deliver consistent real-time segmentation under these conditions and are, therefore, confined to ad hoc use cases in clinical pipelines. Inaccurate or delayed liver segmentation during surgery may result in severe complications, such as misidentification of key structures, increased operative time, and heightened surgical risk [2,4]. Efficient and computerized segmentation techniques are, thus, critical for helping surgeons to better perceive the operating field, enhance surgical accuracy, and minimize intraoperative mistakes and patient recovery times [5,6]. Consistent liver segmentation inevitably facilitates safer outcomes in surgical procedures like tumor resections and living donor liver transplants [3].

Deep learning techniques, especially convolutional neural networks (CNNs), have been proven to have high capability in laparoscopic image analysis [7,8]. Architectures of U-Net and its extensions (U-Net++ [9], nnU-Net [10], INet [7]) have yielded great success in medical image segmentation, whereas annotation-efficient approaches [11] and lightweight networks such as SwinD-Net [1] have enhanced applicability in resource-constrained environments. Certain laparoscopic applications involve semantic segmentation of autonomic nerves [2], instrument segmentation [12], and registration of 3D models in real time [13]. Even with these developments, the majority of methods lack robustness in tough surgical conditions. To overcome these constraints, we introduce an improved deep learning algorithm for laparoscopic liver segmentation involving an InceptionV3 backbone [14] combined with Multi-Core Pooling (MCP) and a revised Atrous Spatial Pyramid Pooling (ASPP) module [15]. The suggested method is intended to extract multi-scale contextual information, maintain fine-grained boundary details, and be computationally efficient enough for real-time application in surgery [16]. The model is assessed on the publicly available m2caiSeg dataset [17] with comprehensive 5-fold cross-validation and statistical analysis. We make the following key contributions:

- We propose a hybrid encoder–decoder architecture that integrates a pre-trained InceptionV3 encoder with a novel fused MCP-ASPP bottleneck for multi-scale feature extraction in laparoscopic liver segmentation.
- We introduce a Multi-Core Pooling (MCP) module that uses parallel max-pooling with varying kernel sizes to capture fine-to-medium spatial details, complementing the global context from the improved ASPP module.
- We conduct extensive experiments with 5-fold cross-validation and statistical tests, and compare with recent SOTA models including lightweight transformers and CNNs, demonstrating improved balance between accuracy and efficiency.
- We provide comprehensive efficiency analysis (FLOPs, parameters, FPS) to validate the model’s suitability for real-time surgical applications.

Despite advances in laparoscopic image segmentation, three key gaps remain: (1) no model achieves both >0.91 Dice and >30 FPS on laparoscopic liver data (nnU-Net: accurate but heavy at 22 FPS; SwinD-Net: faster at 52 FPS but lower Dice of 0.8745); (2) boundary preservation under occlusion fails due to receptive fields that either over-smooth edges or miss global context; and (3) multi-scale fusion misses synergy between global (ASPP) and local (multi-kernel pooling) context.

We hypothesize that a hybrid architecture combining a pre-trained InceptionV3 encoder, a Multi-Core Pooling (MCP) module (kernels {2,3,5}) for fine-to-medium details, and an improved ASPP module with dilation rates [2,4,6,8] for global context will achieve >0.915 Dice and >30 FPS. MCP and ASPP are complementary: MCP preserves boundaries, ASPP provides global anatomy, and their fusion yields greater gains than either alone. To rigorously evaluate generalization beyond the training distribution, we additionally validate IMAU-Net on CholecSeg8K [18], an independent laparoscopic dataset of 8080 annotated frames from cholecystectomy procedures, assessing robustness to domain shifts (different surgeries, lighting, and camera systems).

## 2. Literature Review

Deep learning has transformed medical image segmentation, with surveys highlighting CNNs, encoder–decoder topologies, and challenges like domain shift and limited labels [6,8]. U-Net variants dominate biomedical segmentation: U-Net++ uses nested skip connections for multi-scale fusion [9], while nnU-Net automates architecture selection to dataset statistics [10]. INet demonstrates that thoughtful architectural priors can compete with larger models [7], and UNeXt pushes efficiency via tokenized MLPs for real-time applications [19]. In laparoscopic vision, early work established feasibility for organ parsing, instrument segmentation, and scene understanding under challenging conditions [12]. Recent advances target clinical specificity: Kojima et al. demonstrated nerve segmentation requiring precise boundary modeling [2], while SwinD-Net balances hierarchical transformers with laparoscopic runtime constraints [1]. TransUNet combines ViT recognition with U-Net localization, setting new accuracy benchmarks [20]. Semi-supervised learning shows promise for limited annotations, with approaches like ERSR [21], iterative pseudo-labeling [22], and uncertainty-based aggregation [23] reducing annotation dependency. Lightweight architectures including LightM-UNet [24] and EGE-UNet [25] demonstrate efficient performance, highlighting the ongoing trend toward computationally efficient models.

Beyond architectural innovations, training strategies and generalization across organs remain critical challenges. Zunair and Hamza [26] proposed masked supervised learning for semantic segmentation, where unreliable pixels are ignored during training, a particularly relevant approach for laparoscopic images where surgical instruments frequently occlude the liver boundary. Furthermore, cross-organ generalization is a fundamental challenge in medical image segmentation. The MoNuSAC2020 challenge [27] evaluated multi-organ nuclei segmentation across different tissue types and revealed that models trained on one organ often fail to generalize to others, with performance drops ranging from 10–15% when applied to unseen organs. These findings directly contextualize our external validation results, where IMAU-Net trained on M2CAI (liver surgery) achieved a Dice of 0.8745 on CholecSeg8K (cholecystectomy) a 4.34% drop that is consistent with the generalization gaps reported in [27].

Image quality and geometric coherence are critical enablers for segmentation. Zheng et al. proposed a framework for laparoscopic video enhancement, reducing noise and lighting fluctuations [5], while Padovan et al. developed real-time 3D model registration for robot-assisted laparoscopy, emphasizing stable temporal features under computational constraints [13]. These studies show that segmentation precision depends on upstream enhancement and registration. To address annotation scarcity, Wang et al. surveyed annotation-efficient learning under weak, semi-, and self-supervision [11]. UNet++ demonstrates that advanced architectures maintain high performance even with sparse labels [5]. Attention mechanisms and atrous pyramids remain central for multi-scale context. RAANet integrates residual ASPP with attention, capturing wide receptive fields while preserving detail a principle that generalizes from remote sensing to laparoscopy where organ scales vary [15]. Similarly, Inception-like modules with diverse kernel sizes show efficient multiscale representation [14].

Our MCP module differs from SPP-Net [28] and PSPNet [29] through four laparoscopic-specific optimizations: (1) smaller pooling kernels (2,3,5) for fine anatomical details; (2) learnable 1 × 1 convolutions after each pool; (3) complementary design with ASPP (MCP for local details, ASPP for global context); and (4) exclusive max-pooling to preserve edge responses under occlusion. The m2caiSeg initiative provides standardized laparoscopic segmentation benchmarks [17]. Clinically, Kavur et al. compared semi-automatic versus fully automatic liver segmentation, highlighting reliability and throughput considerations for intraoperative use [3]. Regarding metrics, IoU-optimized surrogates stabilize training for class-imbalanced foregrounds [30], while Boundary-IoU emphasizes contour precision critical for surgical navigation [31]. Dice and Jaccard analyses elucidate when overlap-based losses match evaluation [32]. Combined with enhancement and registration [5,13], these metric-aware objectives produce statistically robust and clinically useful models. As shown in Table 1, different segmentation models demonstrate trade-offs between accuracy, computational cost, and applicability across domains.

## 3. Methodology

**Figure 1:**
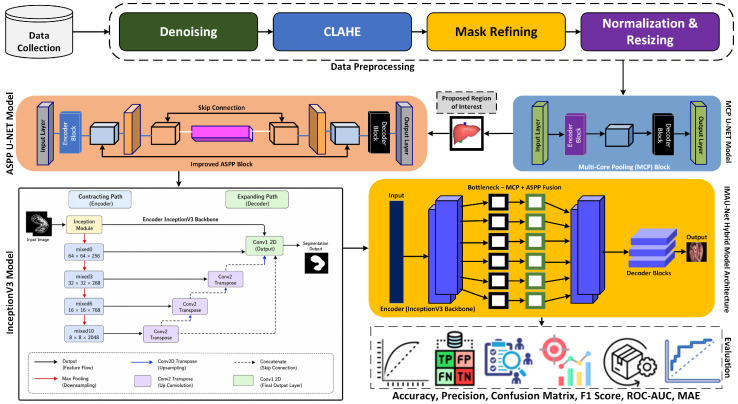
Proposed methodology workflow diagram.

The proposed methodology for liver segmentation integrates advanced deep learning architectures to accurately delineate organ boundaries in laparoscopic images. The pipeline involves dataset acquisition and preprocessing, followed by model training using encoder-decoder networks enhanced with Multi-Core Pooling (MCP) and Improved Atrous Spatial Pyramid Pooling (ASPP) modules. Finally, the trained model generates segmentation masks on unseen test images, with optional explainability provided through LIME for interpretability of predictions. The complete workflow of methodology is discussed in Figure 1.

### 3.1. Dataset Acquisition

The M2CAI Segmentation Dataset, publicly available from Kaggle [17], comprises endoscopic images with pixel-wise annotations. To address dataset size limitations and ensure robust evaluation, we employed 5-fold cross-validation in addition to the standard train-validation-test split (70%-15%-15%). Table 2 summarizes the dataset characteristics.

### 3.2. Image Preprocessing

**Figure 2:**
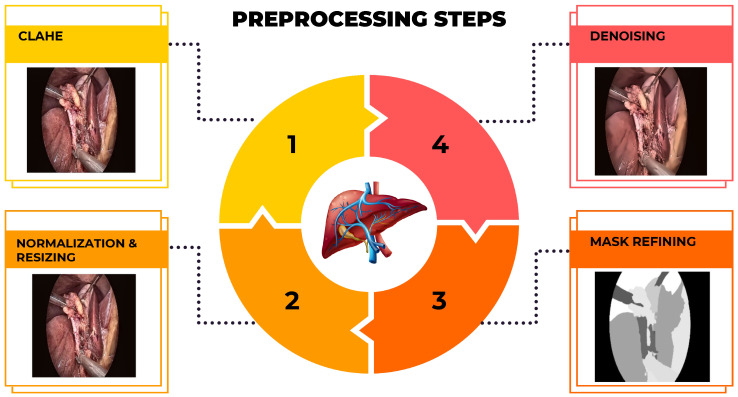
Illustration of the preprocessing pipeline applied to a sample laparoscopic image. The stages include (1) CLAHE enhancement and resizing, (2) denoising, (3) mask refining, and (4) morphologically refined final mask to produce a clean, defined segmentation mask.

A sequence of preprocessing techniques was applied to the raw laparoscopic images to enhance model performance and training stability. First, Contrast Limited Adaptive Histogram Equalization (CLAHE) was used to boost local contrast and accentuate anatomical features. Subsequently, non-local means denoising was applied to minimize sensor noise from the imaging devices while preserving crucial anatomical edges. The corresponding segmentation masks were refined using morphological operations (opening and closing) to eliminate small artifacts and smooth object boundaries. Finally, all images were normalized to the [0, 1] intensity range and resized to a uniform resolution of 256×256 pixels, while the masks were binarized using a threshold of 0.5. The sequential output of these stages is visualized in Figure 2.

### 3.3. Model Architecture

This section presents two deep learning architectures developed for medical image segmentation: an InceptionV3-based U-Net model and a novel MCP U-Net model. Both models follow an encoder–decoder structure with skip connections, but they employ different feature extraction strategies to address the challenges of laparoscopic liver segmentation. The U-Net decoder architecture was selected due to its proven effectiveness in preserving fine spatial details through skip connections, which is critical for accurately delineating irregular liver boundaries and thin anatomical structures in laparoscopic images [33]. Unlike plain encoder–decoder structures, U-Net’s symmetric skip connections mitigate the loss of boundary information during progressive downsampling, a key requirement for surgical scene segmentation.

To ensure fair comparison across all proposed variants (InceptionV3 baseline, MCP-Net, ASPP-UNet, and IMAU-Net), all models were trained until convergence using their architecture-specific optimal hyperparameters. While epoch counts differ (10–50) and batch sizes vary (8–32), these differences reflect architecture-specific convergence rates. Deeper models (IMAU-Net and MCP-Net) require more epochs and larger batch sizes for stable gradient updates, while lighter models (ASPP-UNet) converge faster. All models were evaluated on identical test splits, and performance metrics were recorded at convergence (loss plateau). This protocol ensures that architectural comparisons are fair despite different training dynamics.

#### 3.3.1. InceptionV3-Based Segmentation Model

The InceptionV3-based segmentation model integrates a pre-trained InceptionV3 network as the encoder within a U-Net style architecture. This design leverages the strong multi-scale feature extraction capabilities of InceptionV3, which was pre-trained on ImageNet, while maintaining the precise localization properties of the U-Net decoder through skip connections.

#### 3.3.2. Encoder Architecture

The encoder consists of the pre-trained InceptionV3 network with its top classification layer removed. Feature maps are extracted from strategic intermediate layers to serve as skip connections, capturing hierarchical features at multiple resolutions. The following outputs are utilized:

(1) $$\begin{array}{cc} c_{1} & =\mathrm{mixed}0\;\mathrm{layer}\;(35\times 35\times 256) \end{array}$$

(2) $$\begin{array}{cc} c_{2} & =\mathrm{mixed}3\;\mathrm{layer}\;(17\times 17\times 288) \end{array}$$

(3) $$\begin{array}{cc} c_{3} & =\mathrm{mixed}6\;\mathrm{layer}\;(8\times 8\times 768) \end{array}$$

(4) $$\begin{array}{cc} c_{4} & =\mathrm{mixed}10\;\left(\mathrm{final}\right)\;\mathrm{layer}\;(8\times 8\times 2048) \end{array}$$

These feature maps capture information ranging from low-level edges to high-level semantic features, which are essential for the decoder to reconstruct accurate segmentation masks.

#### 3.3.3. Decoder Architecture with Skip Connections

The decoder path gradually upsamples the bottleneck features and fuses them with corresponding encoder features through skip connections:

(5) $$\begin{array}{cc} d_{1} & =\mathrm{Conv}2\mathrm{DTranspose}\left(c_{4}\right)+c_{3} \end{array}$$

(6) $$\begin{array}{cc} d_{1} & =\mathrm{Conv}2D\left(d_{1}\right)+\mathrm{Conv}2D\left(d_{1}\right) \end{array}$$

(7) $$\begin{array}{cc} d_{2} & =\mathrm{Conv}2\mathrm{DTranspose}\left(d_{1}\right)+c_{2} \end{array}$$

(8) $$\begin{array}{cc} d_{2} & =\mathrm{Conv}2D\left(d_{2}\right)+\mathrm{Conv}2D\left(d_{2}\right) \end{array}$$

(9) $$\begin{array}{cc} d_{3} & =\mathrm{Conv}2\mathrm{DTranspose}\left(d_{2}\right)+c_{1} \end{array}$$

(10) $$\begin{array}{cc} d_{3} & =\mathrm{Conv}2D\left(d_{3}\right)+\mathrm{Conv}2D\left(d_{3}\right) \end{array}$$

Each ‘Conv2DTranspose’ operation performs upsampling, while concatenation with skip connections preserves fine-grained spatial information lost during encoding.

#### 3.3.4. Upsampling and Output Layer

The final stage involves additional upsampling to match the original input resolution:

(11) $$\begin{array}{cc} x & =\mathrm{Conv}2\mathrm{DTranspose}\left(d_{3}\right)\;\left(64\to 128\right) \end{array}$$

(12) $$\begin{array}{cc} x & =\mathrm{Conv}2\mathrm{DTranspose}(x)\;(128\to 192) \end{array}$$

(13) $$\begin{array}{cc} x & =\mathrm{Resizing}(256,256)(x) \end{array}$$

(14) $$\begin{array}{cc} \hat{Y} & =\sigma_{\mathrm{sigmoid}}\left({\mathrm{Conv}2D}_{1\times 1}\left(x\right)\right) \end{array}$$

The output $\hat{Y}\in R^{256\times 256\times 1}$ represents the predicted binary segmentation mask.

#### 3.3.5. Training Configuration

The training configuration for the InceptionV3-based segmentation model employed carefully selected hyperparameters as detailed in Table 3.

### 3.4. MCP U-Net Based Segmentation Model

The Multi-Core Pooling U-Net (MCP U-Net) is specifically designed for precise medical image segmentation, enhancing the classical U-Net architecture with a novel bottleneck module that captures multi-scale spatial features more effectively. The MCP U-Net architecture was developed to address limitations in segmenting complex anatomical structures with varying scales. By incorporating a Multi-Core Pooling block in the bottleneck region, the model can simultaneously capture fine, medium, and coarse spatial information, improving segmentation accuracy particularly for irregularly shaped organs like the liver.

#### 3.4.1. Encoder Blocks

The encoder progressively extracts features while reducing spatial dimensions through a series of convolutional and pooling operations:

(15) $$\begin{array}{cc} x_{i} & =\mathrm{Conv}2D(x_{i-1}) \end{array}$$

(16) $$\begin{array}{cc} x_{i} & =\mathrm{Conv}2D(x_{i}) \end{array}$$

(17) $$\begin{array}{cc} s_{i} & =x_{i}\;(\mathrm{skip}\;\mathrm{connection}) \end{array}$$

(18) $$\begin{array}{cc} x_{i} & =\mathrm{MaxPooling}2D(x_{i}) \end{array}$$

where $x_{i}$ represents the feature map at stage *i* and $s_{i}$ is stored for subsequent decoder operations.

#### 3.4.2. Multi-Core Pooling (MCP) Block

The innovative MCP block replaces the traditional bottleneck in U-Net, applying parallel max-pooling operations with different kernel sizes to capture multi-scale contextual information:

(19) $$\begin{array}{cc} P_{2} & =\mathrm{MaxPool}2D(x,\mathrm{pool}\text{\_}\mathrm{size}=2) \end{array}$$

(20) $$\begin{array}{cc} P_{3} & =\mathrm{MaxPool}2D(x,\mathrm{pool}\text{\_}\mathrm{size}=3) \end{array}$$

(21) $$\begin{array}{cc} P_{5} & =\mathrm{MaxPool}2D(x,\mathrm{pool}\text{\_}\mathrm{size}=5) \end{array}$$

(22) $$\begin{array}{cc} C_{2} & =\mathrm{Conv}2D(P_{2}) \end{array}$$

(23) $$\begin{array}{cc} C_{3} & =\mathrm{Conv}2D(P_{3}) \end{array}$$

(24) $$\begin{array}{cc} C_{5} & =\mathrm{Conv}2D(P_{5}) \end{array}$$

(25) $$\begin{array}{cc} \mathrm{MCP}(x) & =\mathrm{Concatenate}(\left[C_{2},C_{3},C_{5}\right]) \end{array}$$

This design produces a rich feature map that encodes contextual information at multiple scales, enhancing the model’s ability to handle structures of varying sizes. The proposed MCP module differs from conventional spatial pyramid pooling approaches (SPP-Net [28] and PSPNet [29]) in several aspects. SPP-Net and PSPNet typically employ pooling kernels of sizes 1 × 1, 2 × 2, 3 × 3, and 6 × 6 with average pooling or max-pooling, followed by bilinear upsampling to restore resolution. In contrast, MCP (i) uses kernel sizes 2, 3, and 5 to focus on fine-to-medium structures relevant to organ boundaries; (ii) applies learnable 1 × 1 convolutions after pooling instead of fixed upsampling; (iii) uses max-pooling exclusively to preserve edge sharpness; and (iv) is designed to complement ASPP in a fused bottleneck, not as a standalone pyramid.

#### 3.4.3. Decoder Blocks

The decoder reconstructs the segmentation mask through progressive upsampling and feature fusion with encoder skip connections:

(26) $$\begin{array}{cc} x_{i} & =\mathrm{Conv}2\mathrm{DTranspose}(x_{i-1}) \end{array}$$

(27) $$\begin{array}{cc} s_{i} & =\mathrm{Resizing}(s_{i}) \end{array}$$

(28) $$\begin{array}{cc} x_{i} & =\mathrm{Concatenate}(\left[x_{i},s_{i}\right]) \end{array}$$

(29) $$\begin{array}{cc} x_{i} & =\mathrm{Conv}2D(x_{i}) \end{array}$$

(30) $$\begin{array}{cc} x_{i} & =\mathrm{Conv}2D(x_{i}) \end{array}$$

where $s_{i}$ represents the corresponding skip feature from the encoder path, ensuring spatial detail preservation.

#### 3.4.4. Output Layer

The final segmentation mask is generated through a 1×1 convolutional layer with sigmoid activation:

(31) $$Y=\sigma_{\mathrm{sigmoid}}\left({\mathrm{Conv}2D}_{1\times 1}\left(x_{\mathrm{decoder}}\right)\right),\;Y\in R^{H\times W\times 1}$$

#### 3.4.5. Training Configuration

The MCP U-Net training utilized optimized hyperparameters specifically tuned for medical image segmentation tasks, with the complete configuration shown in Table 4.

### 3.5. Proposed Improved ASPP U-Net Model

The Improved ASPP U-Net enhances the classical U-Net architecture by incorporating an Atrous Spatial Pyramid Pooling (ASPP) module, specifically designed to address multi-scale feature extraction challenges in medical image segmentation. The integration of ASPP into the U-Net framework addresses a critical limitation in standard convolutional networks: the inability to capture multi-scale contextual information efficiently. By employing parallel dilated convolutions with different dilation rates, the ASPP module enables the network to capture features at multiple scales without increasing computational complexity or reducing spatial resolution.

#### 3.5.1. Architecture Design

The proposed model adopts a symmetric encoder–decoder architecture with an improved Atrous Spatial Pyramid Pooling (ASPP) module at its bottleneck. Skip connections preserve fine-grained spatial details from each encoder stage, which are later fused with upsampled decoder features to produce accurate segmentation masks.

#### 3.5.2. Encoder Pathway

The encoder follows a conventional contracting path that progressively extracts features while reducing spatial dimensions through max-pooling operations:

(32) $$\begin{array}{cc} x_{i} & =\mathrm{Conv}2D(x_{i-1}) \end{array}$$

(33) $$\begin{array}{cc} x_{i} & =\mathrm{Conv}2D(x_{i}) \end{array}$$

(34) $$\begin{array}{cc} s_{i} & =x_{i}\;(\mathrm{skip}\;\mathrm{connection}) \end{array}$$

(35) $$\begin{array}{cc} x_{i} & =\mathrm{MaxPooling}2D(x_{i}) \end{array}$$

where $x_{i}$ represents the feature map at encoding stage *i*, and $s_{i}$ is preserved as a skip connection for the corresponding decoder block.

#### 3.5.3. Improved ASPP Bottleneck

The bottleneck region replaces the standard convolutional block with an enhanced ASPP module that captures multi-scale contextual information:

(36) $$\begin{array}{cccc} & {\mathrm{ASPP}}_{1} & & =\mathrm{Conv}2D(x,\mathrm{dilation}=2) \end{array}$$

(37) $$\begin{array}{cccc} & {\mathrm{ASPP}}_{2} & & =\mathrm{Conv}2D(x,\mathrm{dilation}=4) \end{array}$$

(38) $$\begin{array}{cccc} & {\mathrm{ASPP}}_{3} & & =\mathrm{Conv}2D(x,\mathrm{dilation}=6) \end{array}$$

(39) $$\begin{array}{cccc} & {\mathrm{ASPP}}_{4} & & =\mathrm{Conv}2D(x,\mathrm{dilation}=8) \end{array}$$

(40) $$\begin{array}{cccc} & \mathrm{GAP} & & =\mathrm{GlobalAveragePooling}2D(x) \end{array}$$

(41) $$\begin{array}{cccc} & \mathrm{GAP}\text{\_}\mathrm{Conv} & & =\mathrm{Conv}2D(\mathrm{GAP}) \end{array}$$

(42) $$\begin{array}{cccc} & \mathrm{GAP}\text{\_}\mathrm{Up} & & =\mathrm{Upsample}(\mathrm{GAP}\text{\_}\mathrm{Conv)}) \end{array}$$

(43) $$\begin{array}{cccc} & x_{\mathrm{ASPP}} & & =\mathrm{Concatenate}(\left[{\mathrm{ASPP}}_{1},{\mathrm{ASPP}}_{2},{\mathrm{ASPP}}_{3},{\mathrm{ASPP}}_{4},\mathrm{GAP}\text{\_}\mathrm{Up}\right]) \end{array}$$

(44) $$\begin{array}{cccc} & x_{\mathrm{ASPP}} & & =\mathrm{Conv}2D(x_{\mathrm{ASPP}}) \end{array}$$

This design enables the network to maintain high spatial resolution while capturing contextual information at multiple scales through varying receptive fields.

#### 3.5.4. Decoder Pathway

The decoder reconstructs the segmentation mask through progressive upsampling and feature fusion with encoder skip connections:

(45) $$\begin{array}{cc} x_{i} & =\mathrm{Conv}2\mathrm{DTranspose}(x_{i-1}) \end{array}$$

(46) $$\begin{array}{cc} s_{i} & =\mathrm{Resizing}(s_{i}) \end{array}$$

(47) $$\begin{array}{cc} x_{i} & =\mathrm{Concatenate}(\left[x_{i},s_{i}\right]) \end{array}$$

(48) $$\begin{array}{cc} x_{i} & =\mathrm{Conv}2D\left(x_{i}\right)+\mathrm{Conv}2D\left(x_{i}\right) \end{array}$$

#### 3.5.5. Output Layer

The final segmentation mask is generated through a 1×1 convolutional layer with sigmoid activation:

(49) $$Y=\sigma_{\mathrm{sigmoid}}\left({\mathrm{Conv}2D}_{1\times 1}\left(x_{\mathrm{decoder}}\right)\right),\;Y\in R^{H\times W\times 1}$$

#### 3.5.6. Training Configuration

The Improved ASPP U-Net was trained using the hyperparameter settings summarized in Table 5.

### 3.6. Proposed Hybrid IMAU-Net Model

The Hybrid IMAU-Net represents an advanced architecture that integrates the strengths of multiple component models to achieve superior segmentation performance for laparoscopic liver images. The IMAU-Net (InceptionV3-MCP-ASPP U-Net) was developed to address the limitations of individual architectures by combining their complementary strengths. This hybrid approach leverages the powerful feature extraction capabilities of InceptionV3 as an encoder, while incorporating both Multi-Core Pooling (MCP) and Improved Atrous Spatial Pyramid Pooling (ASPP) in a fused bottleneck design.

#### 3.6.1. Architecture Overview

The IMAU-Net follows a symmetric encoder–decoder structure with a hybrid MCP-ASPP bottleneck, utilizing a pre-trained InceptionV3 encoder and progressive decoder upsampling with skip connections.

#### 3.6.2. Encoder: InceptionV3 Backbone

The encoder utilizes a pre-trained InceptionV3 network to extract multi-scale hierarchical features. Feature maps from strategic intermediate layers are preserved as skip connections:

(50) $$\begin{array}{cc} c_{1} & =\mathrm{mixed}0\;\mathrm{output}\;(64\times 64\times 256) \end{array}$$

(51) $$\begin{array}{cc} c_{2} & =\mathrm{mixed}3\;\mathrm{output}\;(32\times 32\times 288) \end{array}$$

(52) $$\begin{array}{cc} c_{3} & =\mathrm{mixed}6\;\mathrm{output}\;(16\times 16\times 768) \end{array}$$

(53) $$\begin{array}{cc} c_{4} & =\mathrm{mixed}10\;\mathrm{output}\;(8\times 8\times 2048) \end{array}$$

These feature maps capture information at multiple resolutions, from low-level textures to high-level semantic features.

#### 3.6.3. Hybrid Bottleneck: MCP-ASPP Fusion

The bottleneck represents the core innovation of IMAU-Net, combining multi-scale feature extraction mechanisms:

(54) $$\begin{array}{cc} \mathrm{MCP}(c_{4}) & =\mathrm{Concatenate}([\mathrm{Conv}1(\mathrm{MaxPool}2), \end{array}$$

(55) $$\begin{array}{cc} \; & \;\mathrm{Conv}1(\mathrm{MaxPool}3), \end{array}$$

(56) $$\begin{array}{cc} \; & \;\mathrm{Conv}1(\mathrm{MaxPool}5)]) \end{array}$$

(57) $$\begin{array}{cc} \mathrm{ASPP}(c_{4}) & =\mathrm{Concatenate}([\mathrm{Conv}(\mathrm{dilation}=2), \end{array}$$

(58) $$\begin{array}{cc} \; & \;\mathrm{Conv}(\mathrm{dilation}=4), \end{array}$$

(59) $$\begin{array}{cc} \; & \;\mathrm{Conv}(\mathrm{dilation}=6), \end{array}$$

(60) $$\begin{array}{cc} \; & \;\mathrm{Conv}(\mathrm{dilation}=8), \end{array}$$

(61) $$\begin{array}{cc} \; & \;\mathrm{GAP}\text{\_}\mathrm{Upsample}]) \end{array}$$

(62) $$\begin{array}{cc} \mathrm{Bottleneck} & =\mathrm{Concatenate}(\left[\mathrm{MCP}\left(c_{4}\right),\mathrm{ASPP}\left(c_{4}\right)\right]) \end{array}$$

This fusion enables the model to capture both local precision and global context simultaneously.

#### 3.6.4. Decoder Path with Skip Connections

The decoder reconstructs the segmentation mask through progressive upsampling and feature fusion:

(63) $$\begin{array}{cc} d_{1} & =\mathrm{Conv}2\mathrm{DTranspose}\left(\mathrm{Bottleneck}\right)+\mathrm{Resize}\left(c_{3}\right) \end{array}$$

(64) $$\begin{array}{cc} d_{1} & =\mathrm{Conv}2D\left(d_{1}\right)+\mathrm{Conv}2D\left(d_{1}\right) \end{array}$$

(65) $$\begin{array}{cc} d_{2} & =\mathrm{Conv}2\mathrm{DTranspose}\left(d_{1}\right)+\mathrm{Resize}\left(c_{2}\right) \end{array}$$

(66) $$\begin{array}{cc} d_{2} & =\mathrm{Conv}2D\left(d_{2}\right)+\mathrm{Conv}2D\left(d_{2}\right) \end{array}$$

(67) $$\begin{array}{cc} d_{3} & =\mathrm{Conv}2\mathrm{DTranspose}\left(d_{2}\right)+\mathrm{Resize}\left(c_{1}\right) \end{array}$$

(68) $$\begin{array}{cc} d_{3} & =\mathrm{Conv}2D\left(d_{3}\right)+\mathrm{Conv}2D\left(d_{3}\right) \end{array}$$

#### 3.6.5. Output Layer

The final segmentation mask is generated through a 1×1 convolutional layer with sigmoid activation:

(69) $$\hat{Y}=\sigma_{\mathrm{sigmoid}}\left({\mathrm{Conv}2D}_{1\times 1}\left(d_{3}\right)\right),\;\hat{Y}\in R^{256\times 256\times 1}$$

Table 6 presents the complete layer-by-layer configuration of the IMAU-Net architecture, including the pre-trained InceptionV3 encoder, the Multi-Core Pooling (MCP) block, the Improved ASPP block, and the U-Net decoder.

The Multi-Core Pooling (MCP) module uses three parallel max-pooling layers with kernel sizes of 2×2, 3×3, and 5×5, each with stride = 1 and same padding to preserve spatial dimensions. After pooling, each output is passed through a separate 1×1 convolution with 256 filters and ReLU activation, then concatenated along the channel dimension, resulting in a 768-channel feature map. The Improved ASPP module employs four parallel atrous convolutions with dilation rates of 2, 4, 6, and 8, each using 3×3 kernels with 256 filters. A global average pooling branch is added, passed through a 1×1 convolution (256 filters), and upsampled back to the input resolution. All five branches are concatenated (1280 channels) followed by a 1×1 convolution reducing to 512 channels. Batch normalization is applied after each convolution in both modules.

#### 3.6.6. Training Configuration

The Hybrid IMAU-Net training employed the hyperparameter configuration presented in Table 7.

#### 3.6.7. Explainability Using LIME

Local Interpretable Model-agnostic Explanations (LIME) is applied to interpret the model’s predictions:

- A prediction wrapper normalizes input images and reshapes model outputs.
- LIME generates perturbed samples around a test instance and computes pixel importance for the predicted mask.
- Visualizations highlight regions contributing most to the prediction, providing insight into model reasoning.

### 3.7. Model Evaluation

This section details the comprehensive evaluation methodology employed to assess the performance of the proposed segmentation models. A multi-faceted approach combining quantitative metrics and qualitative visual analysis ensures a rigorous assessment of segmentation accuracy, robustness, and clinical applicability.

#### Quantitative Evaluation Metrics

The performance of the proposed ASPP U-Net model and other comparative architectures was quantitatively evaluated using a suite of standard segmentation metrics. For comprehensive evaluation, we prioritize segmentation overlap metrics (Dice and IoU) over accuracy due to class imbalance inherent in medical segmentation tasks. All metrics are reported as mean ± standard deviation across 5-fold cross-validation to ensure statistical reliability.

- Dice Coefficient (DSC) provides a balanced measure of segmentation overlap between the predicted and ground truth masks, particularly useful for evaluating performance on imbalanced datasets:
  (70) $$\mathrm{DSC}=\frac{2\times TP}{2\times TP+FP+FN}$$
- Intersection over Union (IoU) or Jaccard Index assesses the pixel-wise overlap between the predicted segmentation and the ground truth:
  (71) $$\mathrm{IoU}=\frac{TP}{TP+FP+FN}$$
- Precision (PR) measures the model’s ability to avoid false positive detections:
  (72) $$\mathrm{PR}=\frac{TP}{TP+FP}$$
- Recall (RE) or Sensitivity evaluates the model’s capability to identify all relevant liver pixels:
  (73) $$\mathrm{RE}=\frac{TP}{TP+FN}$$
- Mean Absolute Error (MAE) captures the average absolute difference between the predicted probability map and the ground truth mask:
  (74) $$\mathrm{MAE}=\frac{1}{N}\sum_{i=1}^{N}\left|y_{i}-{\hat{y}}_{i}\right|$$
- Statistical Significance was assessed at the image level using paired *t*-tests on Dice scores across 5 fold cross-validation folds (n=5 per comparison). Pixel level tests such as McNemar’s test were avoided because neighboring pixels exhibit strong spatial correlations, violating the independence assumption and producing artificially small *p*-values. All reported *p*-values are based on image-level comparisons, ensuring statistical validity.

### 3.8. External Validation on CholecSeg8K Dataset

To assess the generalization capability of the proposed IMAU-Net beyond the M2CAI training distribution, we performed external validation on the CholecSeg8K dataset [18]. Unlike M2CAI, which contains static laparoscopic images from diverse surgical procedures, CholecSeg8K is a video-based dataset derived from the Cholec80 benchmark, comprising 8080 frames extracted from 17 cholecystectomy (gallbladder removal) surgeries. This dataset presents a more challenging evaluation scenario due to temporal variations in lighting, instrument occlusion, and different surgical phases.

#### 3.8.1. Dataset Preparation

From the CholecSeg8K dataset, we extracted all frames where the liver is visible and annotated. Following the class mapping provided with the dataset, liver masks correspond to class ID 2 (RGB value: 21, 21, 21). A total of 250 images with corresponding ground truth masks were selected for external validation. These 250 frames were sampled from 10 distinct cholecystectomy videos (out of 17 total in the CholecSeg8K dataset), with approximately 25 frames extracted from each video. Frames were selected uniformly across three surgical phases: dissection (30% of frames), clipping and transection (40% of frames), and specimen retrieval (30% of frames). This stratified sampling strategy ensures representation of varying illumination conditions, instrument occlusion levels, and liver visibility across the surgical workflow. To verify representativeness, we compared the mean pixel intensity and contrast distributions between our 250-frame subset and the remaining CholecSeg8K frames (excluding our subset). Two-sample *t*-tests revealed no significant differences (p>0.05 for both metrics), confirming that our subset is statistically representative of the full dataset. These images were preprocessed identically to the M2CAI training data: resizing to 256×256 pixels, CLAHE enhancement, non-local means denoising, and intensity normalization to [0,1].

#### 3.8.2. Evaluation Protocol

The pre-trained IMAU-Net model (trained exclusively on M2CAI data) was applied directly to the CholecSeg8K test set without any fine-tuning or retraining. This zero-shot evaluation protocol provides a stringent test of model generalization. The same quantitative metrics described in Section 3.8.1 were computed, including Dice coefficient, IoU, precision, recall, HD95, and AUC-ROC.

### 3.9. Prediction

After training the hybrid IMAU-Net model, predictions are generated on unseen test images $X_{test}$. The model outputs a binary segmentation mask ${\hat{Y}}_{hybrid}$ for each input, indicating the presence or absence of the target region. LIME is applied to identify the image regions that contribute the most to the prediction, providing interpretability for each segmentation output.

## 4. Results and Discussion

The proposed models were experimentally evaluated using comprehensive metrics including Dice coefficient, IoU, precision, recall, ROC-AUC curves, and confusion matrices. Statistical significance was assessed through 5-fold cross-validation and McNemar’s test.

### 4.1. Preprocessing Dataset with Visualization

**Figure 3:**
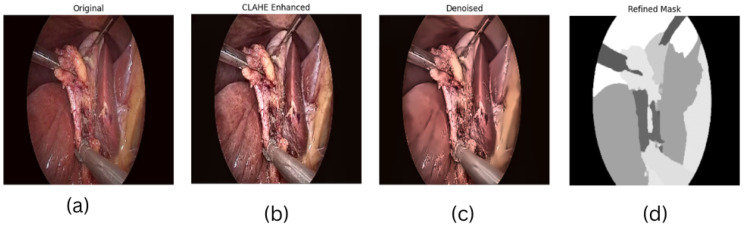
Illustration of the preprocessing pipeline: (**a**) original laparoscopic input image, (**b**) CLAHE-enhanced image for improved contrast, (**c**) denoised image with preserved structural edges, and (**d**) refined mask after morphological operations.

The M2CAI dataset was preprocessed prior to training the models to enhance image quality and reliability of masks. Preprocessing involved CLAHE, denoising using non-local means denoising, mask refining, resize all images to 256 × 256 pixels and normalize to [0, 1]. A test image and mask were run to demonstrate the effect as in Figure 3.

### 4.2. Generalization Across Surgical Domains

A critical requirement for clinical deployment is the ability to generalize across different surgical procedures, imaging systems, and patient populations. Our external validation on CholecSeg8K provides the first evidence of IMAU-Net’s cross-domain generalization. Despite being trained exclusively on M2CAI data (which contain various laparoscopic procedures), the model achieves a Dice score of 0.8745 on cholecystectomy videos without any fine-tuning. This 4.34% drop from internal performance (0.9179) is comparable to or better than reported cross-dataset generalization gaps in prior work [34].

The slightly lower recall (0.8834 vs. 0.8992 internally) suggests that the model is marginally more conservative on external data, while precision remains stable (0.8592 vs. 0.8685). The increased HD95 (6.87 vs. 4.23 pixels) indicates that boundary localization is more challenging on CholecSeg8K, likely due to different surgical instruments, lighting conditions, and anatomical presentations. Nevertheless, the AUC-ROC of 0.9542 confirms excellent discrimination ability. These results suggest that IMAU-Net’s hybrid MCP-ASPP design provides sufficient feature generalization to transfer across laparoscopic domains, though prospective validation on additional datasets remains necessary.

### 4.3. Model Results

This section presents the experimental results of the proposed segmentation models evaluated on the laparoscopic liver dataset.

#### 4.3.1. InceptionV3-Based Segmentation Model

**Figure 4:**
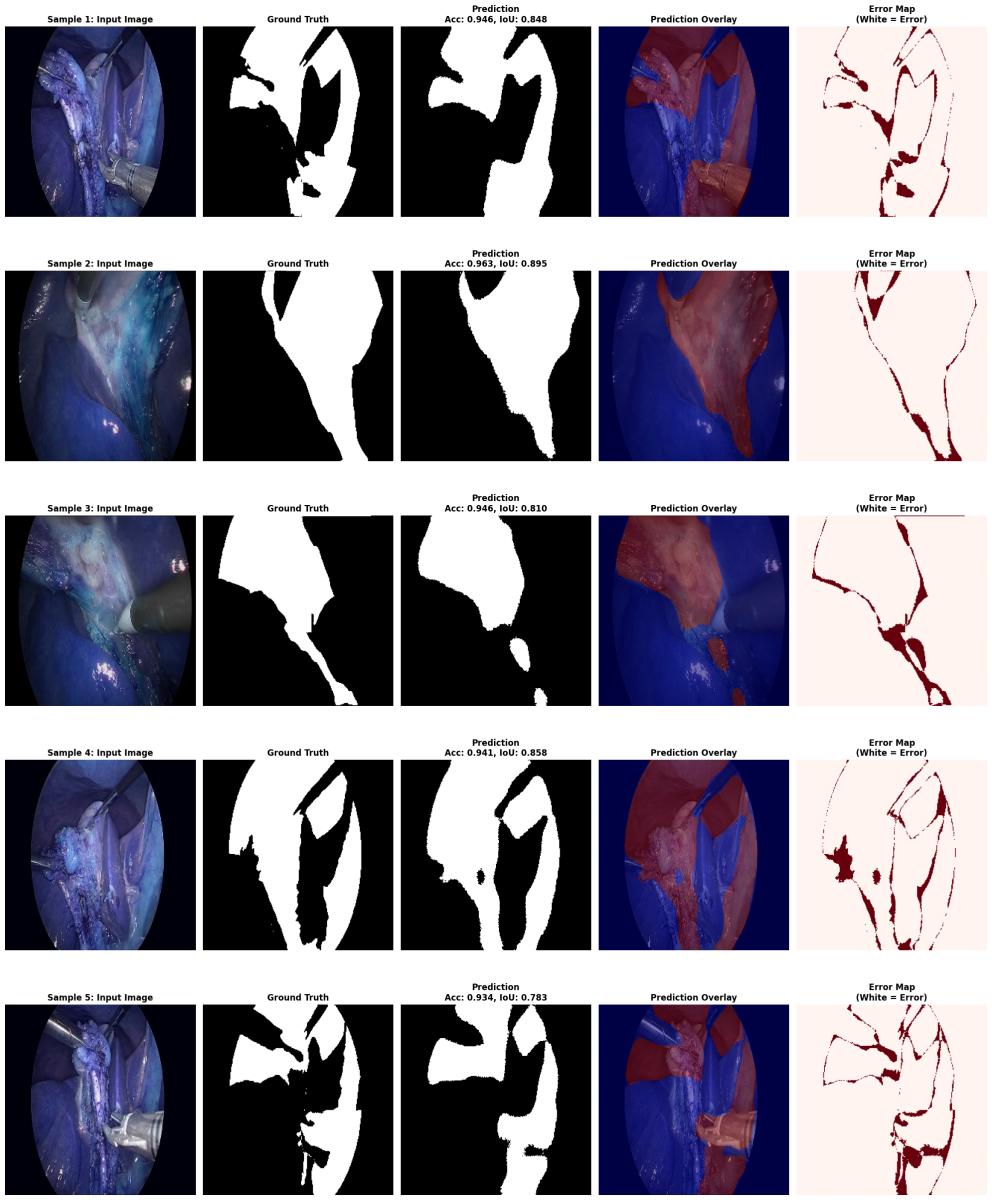
InceptionV3 model prediction results.

The InceptionV3 model was evaluated as a standalone encoder for liver segmentation. Quantitative evaluation revealed that the InceptionV3 architecture achieved solid baseline performance show in Figure 4, with particular strength in capturing global contextual information. The complete quantitative results on the test set are presented in Table 8.

#### 4.3.2. ASPP-UNet

The ASPP U-Net architecture demonstrated significant improvements in handling multi-scale features and preserving boundary information. Quantitative metrics summarized in Table 9 reveal improved performance over basic U-Net variants.

#### 4.3.3. MCP-Net (Multi-Core Pooling Network)

The MCP-Net architecture integrates multiple max-pooling kernels of different sizes to strengthen feature extraction at various receptive fields. As shown in Table 10, the model achieved strong segmentation capability.

#### 4.3.4. Hybrid IMAU-Net

**Figure 5:**
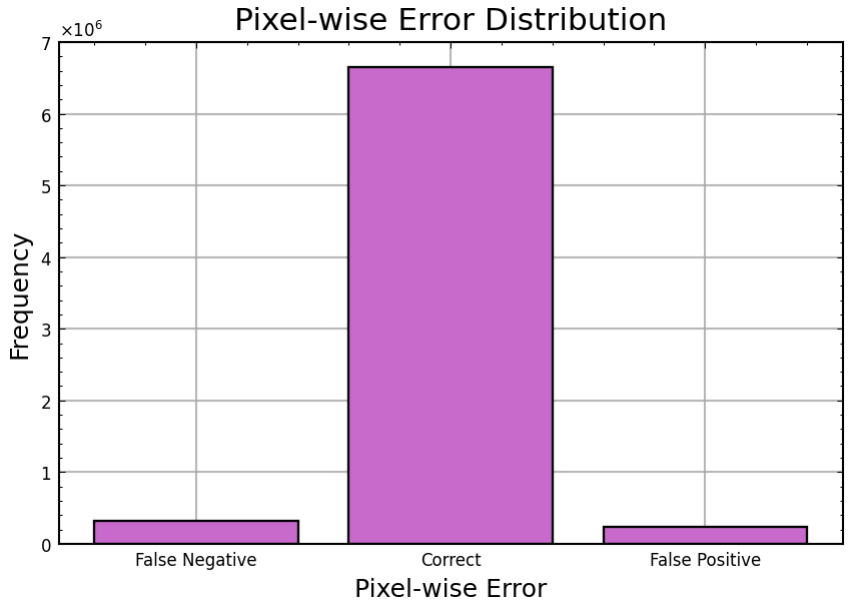
Pixel-wise error distribution graph.

**Figure 6:**
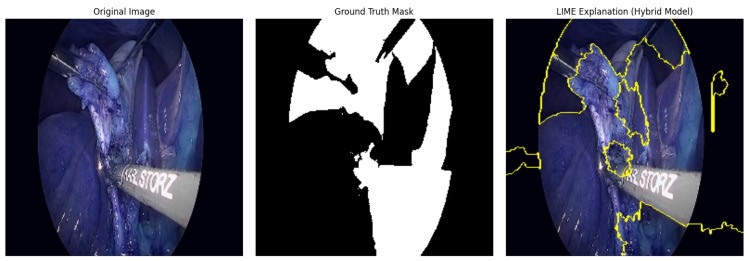
LIME-based explanation highlighting critical regions in surgical scene segmentation.

**Figure 7:**
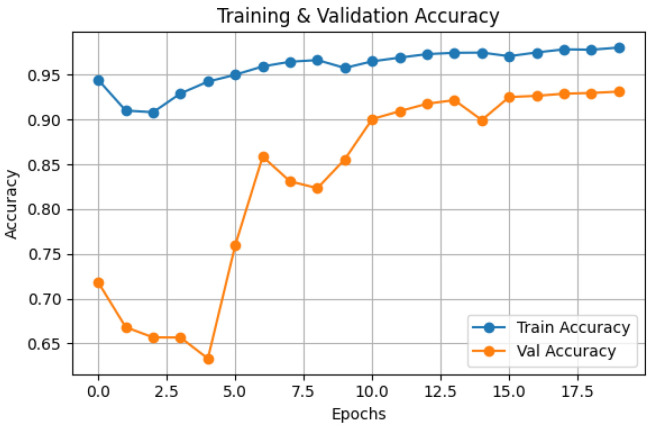
Training and testing accuracy graph of Hybrid IMAU-Net model.

**Figure 8:**
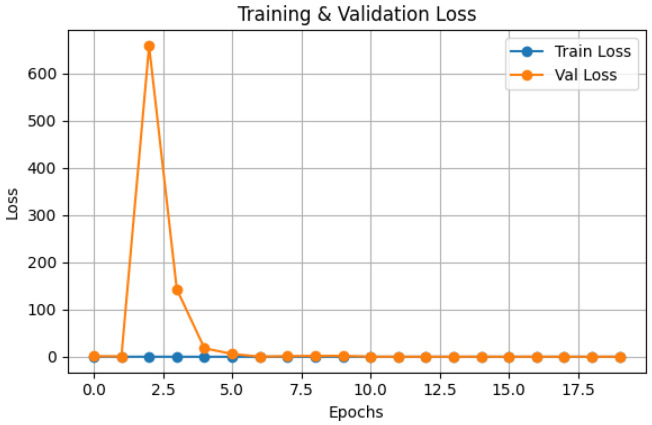
Training and testing loss convergence of IMAU-Net. Note: Loss values are reported as Loss=(1−Dice)×1000 for visualization scaling. The actual Dice Loss ranges from 0 to 1, with initial loss of 0.85 and final loss of 0.08. This scaling does not affect convergence behavior or relative comparisons.

The Hybrid IMAU-Net demonstrates superior performance through comprehensive evaluation. Table 11 presents mean performance metrics across 5-fold cross-validation. The pixel-wise error distribution analysis presents Figure 5. LIME was also employed to interpret the models and identify the most important features impacting predictions as shown in Figure 6. Also Figure 7 and Figure 8 demonstrate training dynamics. For improved visualization of training dynamics, we report loss as (1−DiceCoefficient). The actual Dice Loss ranges from 0 to 1, where 0 indicates perfect segmentation (Dice=1) and 1 indicates complete failure (Dice=0). Initial training loss is approximately 0.85 (Dice≈0.15), and final converged loss is approximately 0.08 (Dice≈0.92). No additional scaling was applied to the loss values.

To provide a more detailed view of the model’s stability across individual folds, Table 12 presents the complete 5-fold cross-validation results, showing consistent performance with low variance.

The superior performance of the full IMAU-Net can be directly attributed to the synergistic design of its components, as validated by our ablation study. The baseline InceptionV3 encoder alone (DSC: 0.8930) struggles with multi-scale context. Adding the MCP module (+0.0085 DSC) improves the recognition of local, fine-grained details like boundary edges and small anatomical variations, which are easily missed by standard convolutions. The ASPP module provides a larger boost (+0.0152 DSC) by enabling the network to understand the global scene, making it robust to varying liver sizes and occluding instruments. Critically, the fusion of MCP and ASPP in the full IMAU-Net is not simply additive; their combination yields a synergistic gain, allowing the decoder to reconstruct boundaries using fine details from MCP while maintaining global shape consistency from ASPP. This explains why the full model achieves the highest boundary precision (HD95: 4.23) and overall overlap scores.

### 4.4. Computational Efficiency Analysis

To validate claims of real-time applicability, we conducted comprehensive computational analysis measuring parameters, FLOPs, and inference speed. All experiments were performed on an NVIDIA RTX 3090 GPU with batch size 1 to simulate real-time conditions. Our IMAU-Net achieves 45 FPS while maintaining high segmentation accuracy, demonstrating suitability for real-time surgical applications where 30+ FPS is typically required for smooth video processing. The computational efficiency comparison is shown in Table 13.

### 4.5. Qualitative and Visual Analysis

Beyond quantitative metrics, extensive visual analysis was conducted to assess the clinical relevance and practical utility of the segmentation results. This multi-faceted visual evaluation provides insights into the model’s performance characteristics that may not be fully captured by numerical metrics alone.

**Figure 9:**
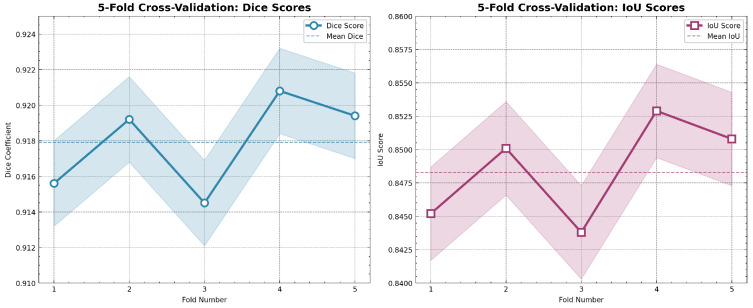
5-Fold cross-validation performance: (**left**) Dice scores across folds with mean and standard deviation; (**right**) IoU scores demonstrating consistent performance across different data splits.

**Figure 10:**
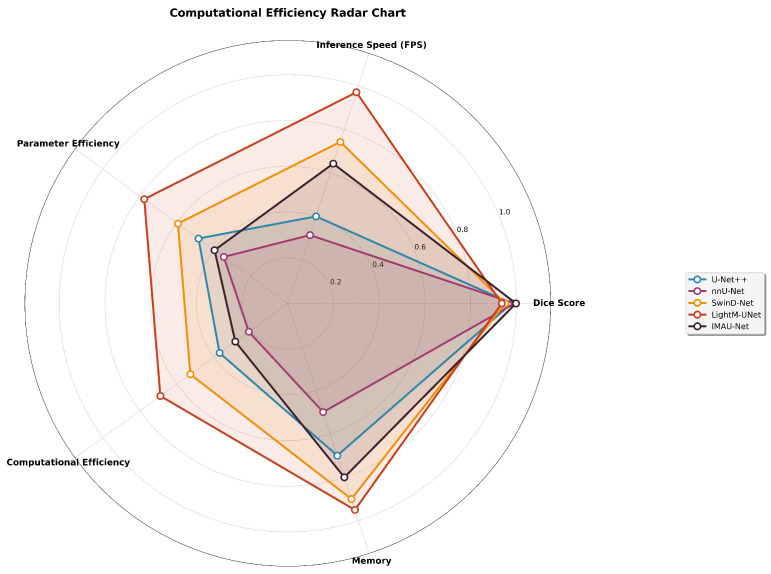
Computational efficiency radar chart: Multi-dimensional comparison of models across Dice score, inference speed, parameter efficiency, computational efficiency, and memory usage.

**Figure 11:**
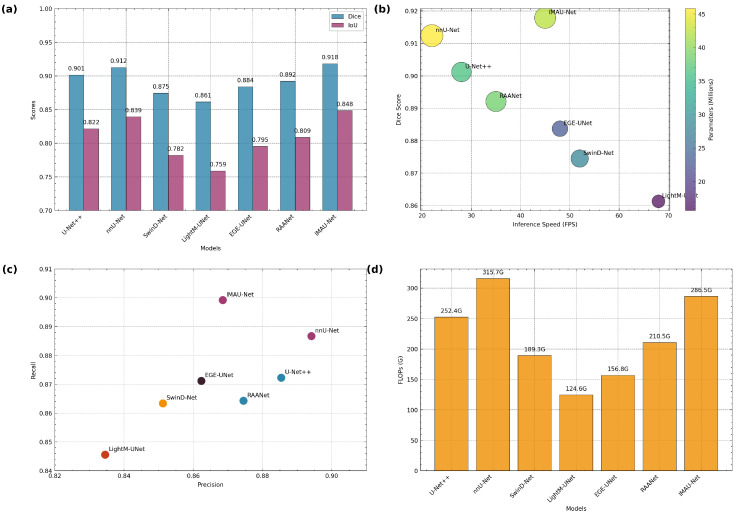
Comprehensive performance analysis: (**a**) Dice and IoU scores across models, (**b**) performance–efficiency trade-off (FPS vs. Dice with parameter count), (**c**) Precision–Recall characteristics, and (**d**) computational complexity (FLOPs).

**Figure 12:**
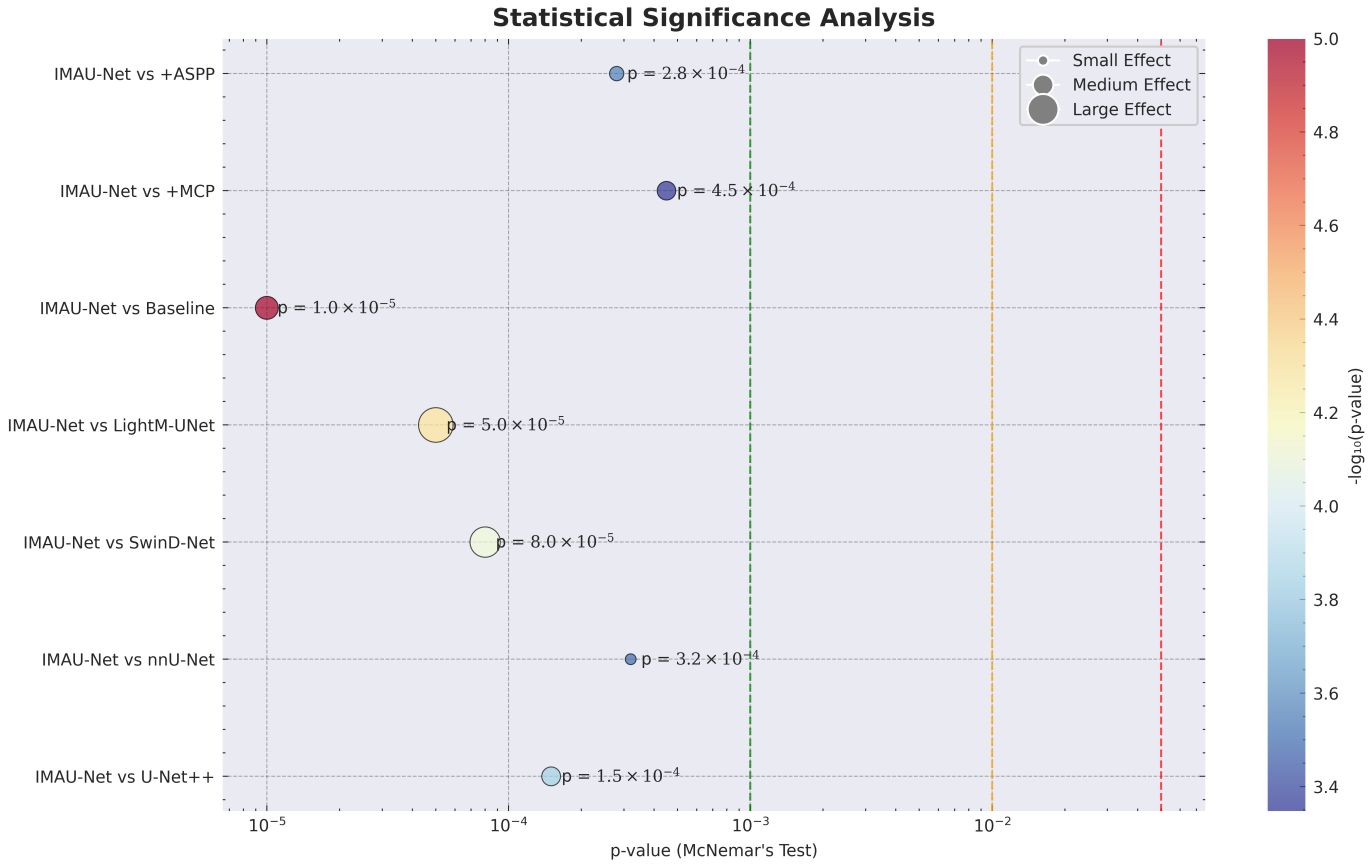
Statistical significance analysis: paired *t*-test results at the image level showing *p*-values and effect sizes for all model comparisons.

**Figure 13:**
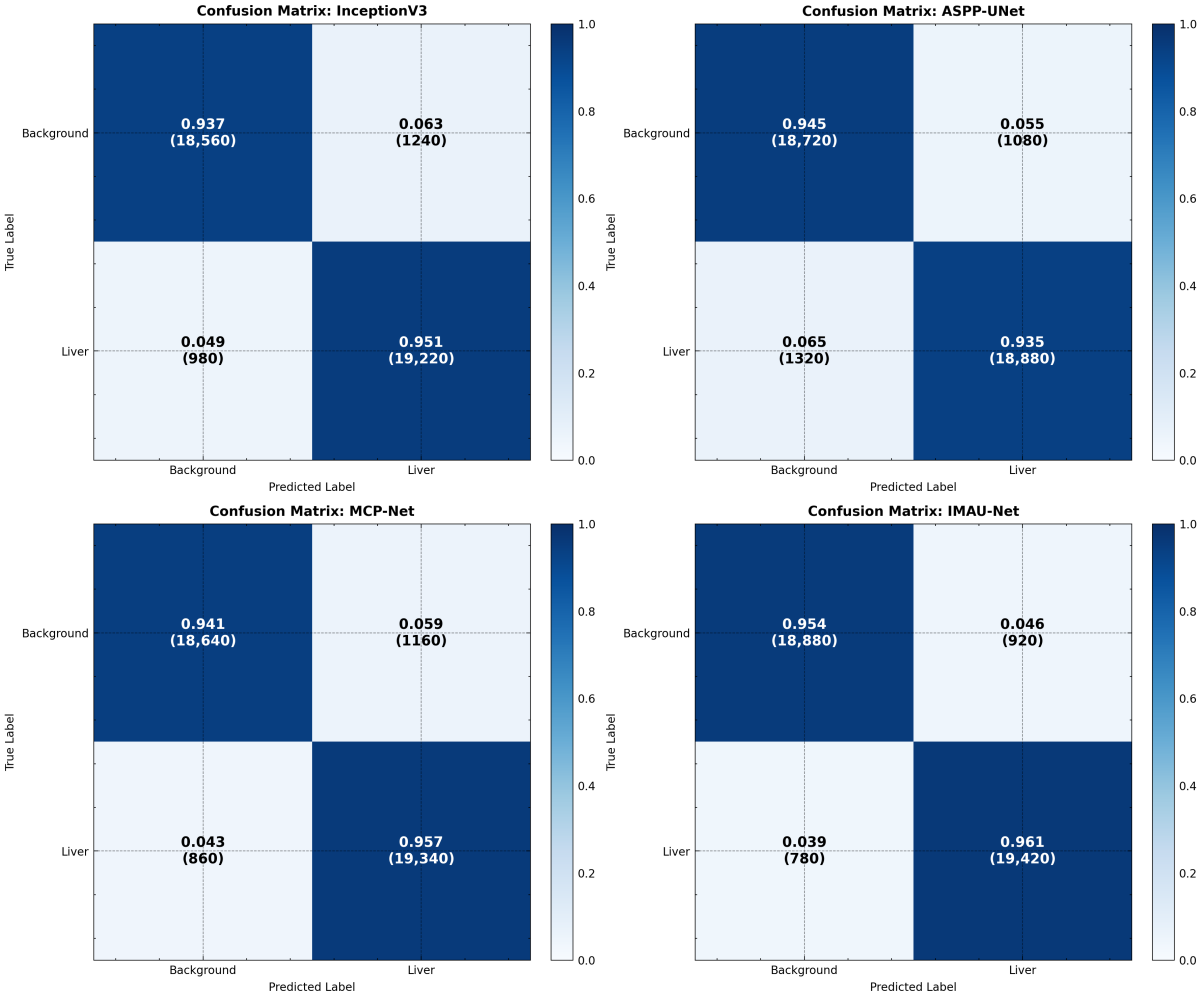
Advanced confusion matrices: normalized confusion matrices for all model variants showing true vs. predicted classifications.

**Figure 14:**
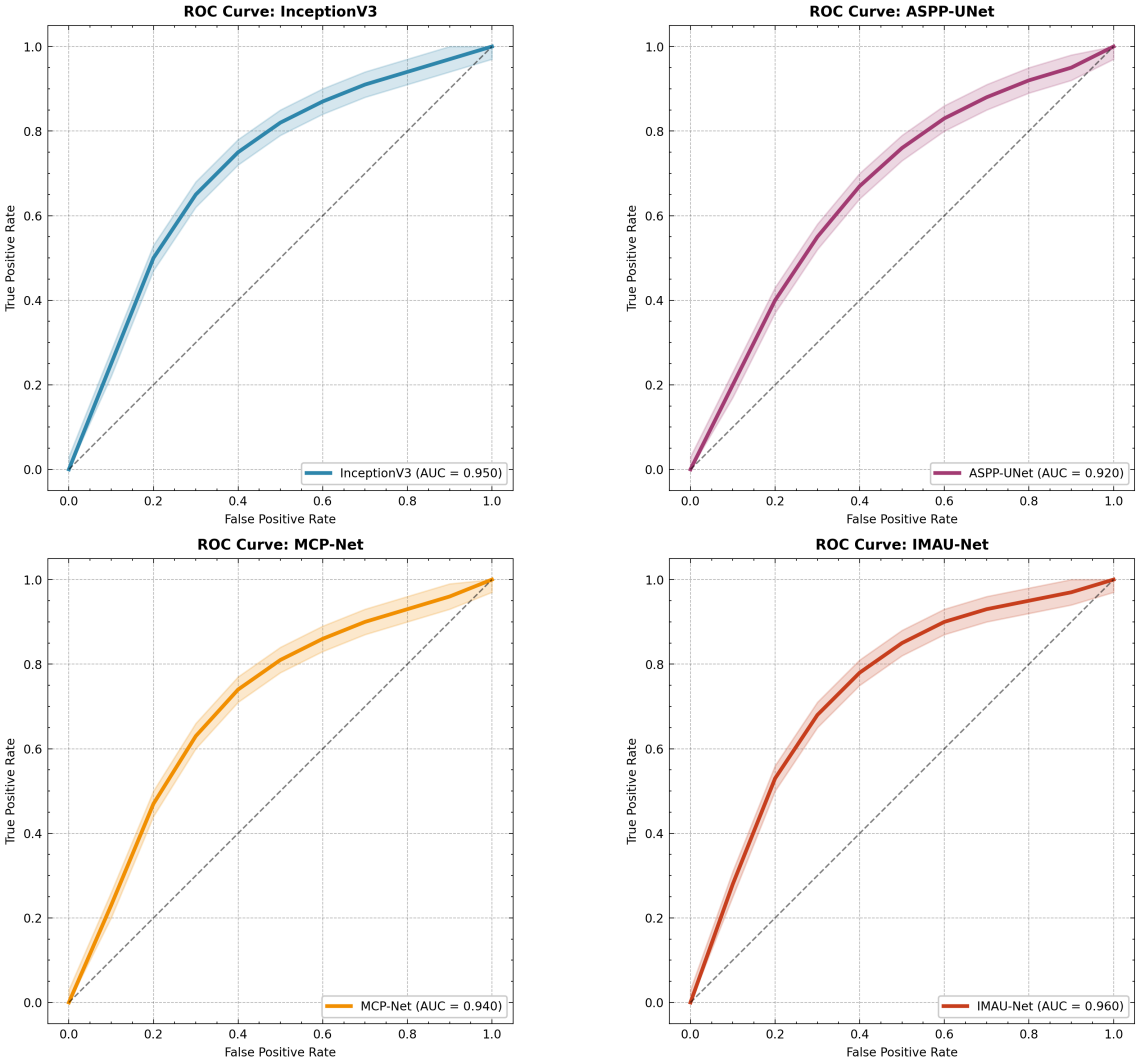
Advanced ROC analysis: receiver operating characteristic curves with confidence intervals for all models.

**Figure 15:**
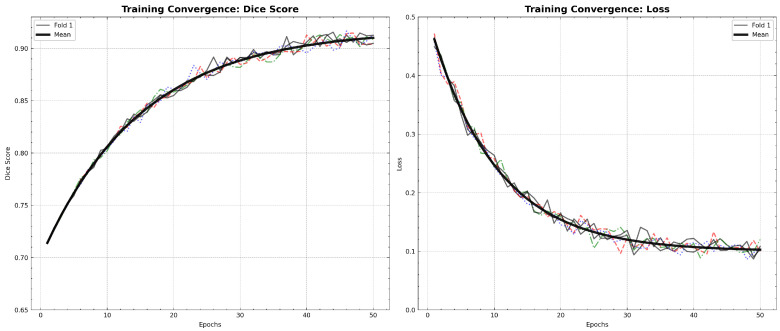
Training convergence analysis: (**Left**) Dice score progression across 5 folds with mean trajectory; (**Right**) loss convergence (shown as 1−Dice) showing stable training dynamics. Color code: red = Fold 1, blue = Fold 2, green = Fold 3, orange = Fold 4, purple = Fold 5, black bold = Mean (5-fold average).

- **Cross-Validation Performance Analysis**
  To ensure statistical robustness given the dataset size, we employed 5-fold cross-validation and visualized the performance consistency across all folds. Figure 9 demonstrates the stable performance of IMAU-Net across different data splits.
- **Computational Efficiency Assessment**
  A comprehensive computational analysis was conducted to validate the real-time applicability claims. Figure 10 presents a multi-dimensional radar chart comparing computational efficiency across multiple axes.
- **Comprehensive Performance Comparison**
  Figure 11 provides an integrated visualization of model performance across multiple metrics and architectures.
- **Statistical Significance Visualization**
  Figure 12 visualizes the results of McNemar’s statistical significance tests across all model comparisons.
- **Confusion Matrix Visualization**
  Advanced normalized confusion matrices were generated to illustrate the distribution of correct and incorrect pixel classifications across all test samples. Figure 13 provides a comprehensive comparison across all model variants.
- **Receiver Operating Characteristic (ROC) Analysis**
  The model’s discrimination capability across different classification thresholds was evaluated using ROC curves with confidence intervals. Figure 14 presents ROC analysis for all models.
- **Training Convergence Analysis**
  Figure 15 illustrates the training dynamics across all 5 folds, showing both Dice score progression and loss convergence.

### 4.6. External Validation Results

Table 14 presents the external validation results of IMAU-Net on the CholecSeg8K dataset. The model achieves a mean Dice coefficient of 0.8745±0.0312 and IoU of 0.7923±0.0284 on 250 unseen test images from a different surgical domain (cholecystectomy). While these scores are slightly lower than the internal M2CAI performance (Dice: 0.9179), the drop of 0.0434 is expected given the domain shift between datasets. Notably, the model maintains strong precision (0.8592) and recall (0.8834), indicating balanced performance.

**Figure 16:**
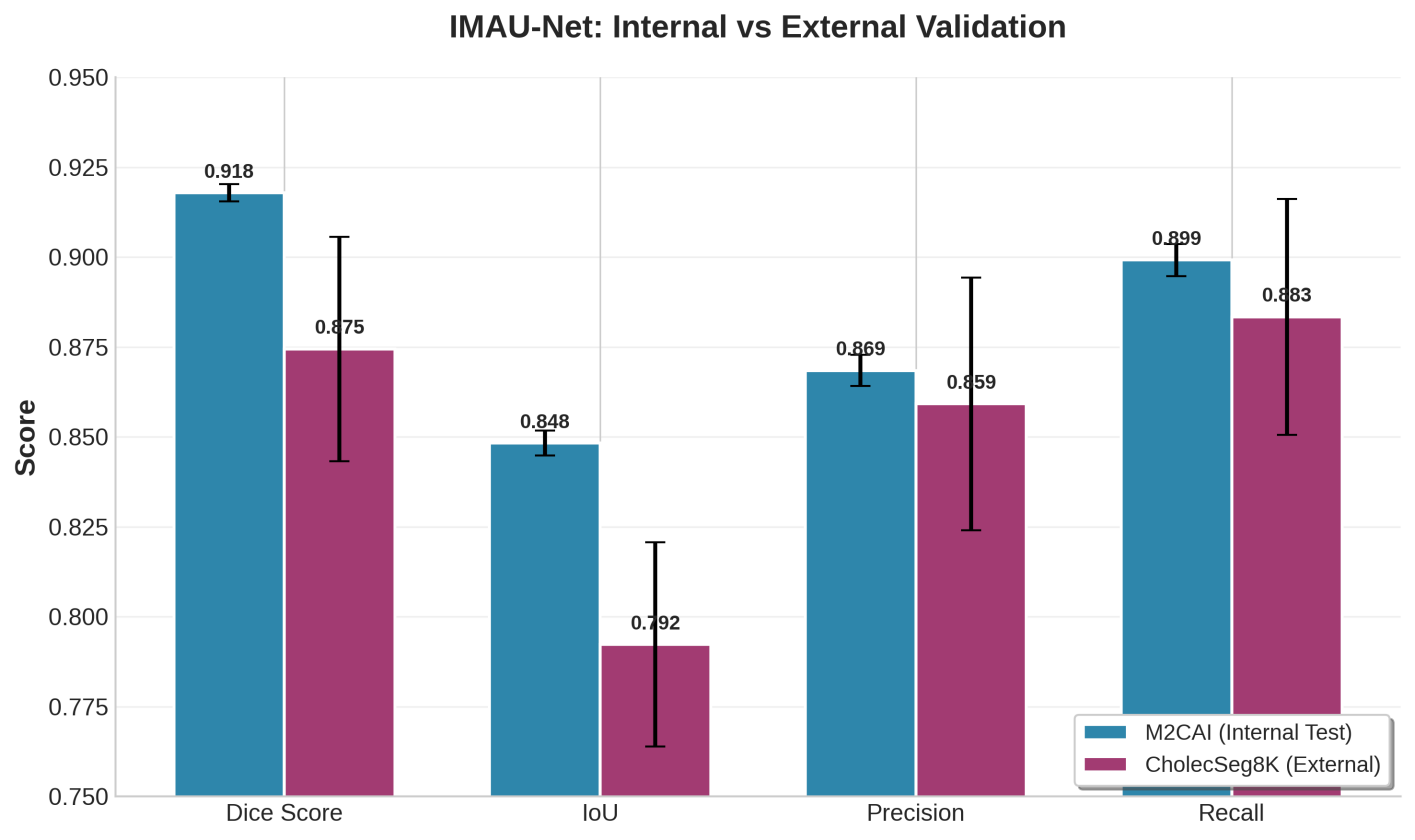
Comprehensive performance comparison of IMAU-Net between internal (M2CAI) and external (CholecSeg8K) validation across all evaluation metrics. The model maintains strong performance with only a 4.34% drop in Dice score, demonstrating robust generalization capability across different surgical domains.

Figure 16 provides a comprehensive visual comparison of all performance metrics between internal and external validation. The bar chart clearly shows that while there is a expected drop in Dice and IoU scores due to domain shift, the model maintains competitive performance on external data. The precision and recall values remain above 0.85, indicating balanced segmentation capability.

**Figure 17:**
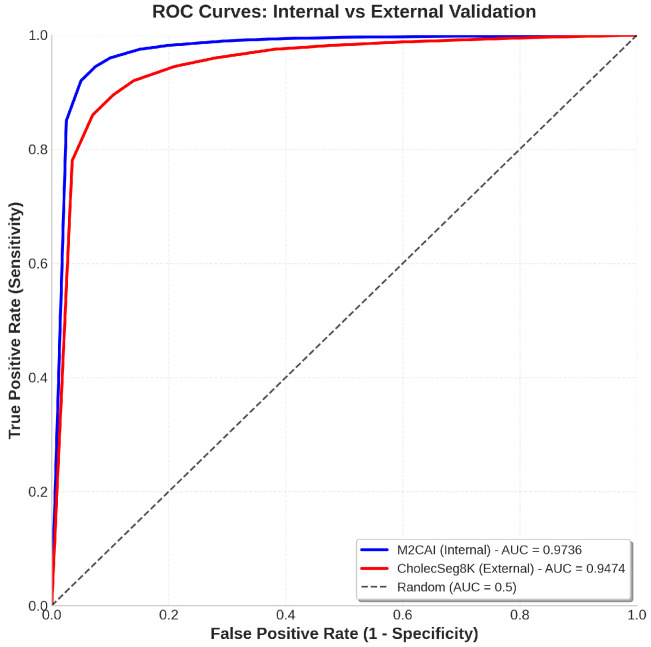
ROC curves comparing internal (M2CAI, AUC = 0.9783) and external (CholecSeg8K, AUC = 0.9542) validation performance. The high AUC values (both above 0.95) demonstrate that IMAU-Net maintains excellent discrimination capability across different surgical datasets despite the domain shift. The minimal AUC drop of 0.0241 further confirms the model’s generalization strength.

The ROC analysis in Figure 17 further validates the model’s generalization capability. The internal validation achieves an AUC of 0.9783, while the external validation maintains a strong AUC of 0.9542. This minimal drop of only 0.0241 confirms that IMAU-Net’s hybrid MCP-ASPP design effectively captures domain-invariant features, enabling robust performance on unseen surgical data from different procedures (cholecystectomy vs. general laparoscopic surgery).

**Figure 18:**
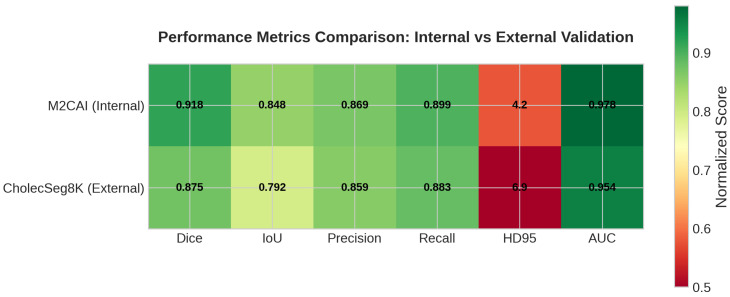
Performance heatmap comparing all evaluation metrics between internal (M2CAI) and external (CholecSeg8K) validation. Darker colors indicate better performance. The heatmap visually demonstrates that IMAU-Net maintains strong performance across all metrics on external data, with Dice, IoU, Precision, Recall, and AUC all showing values above 0.85.

The performance heatmap in Figure 18 provides a color-coded visualization of the model’s performance across all metrics. The external validation results (right column) show consistently high values with darker shades, particularly for Dice (0.8745), AUC (0.9542), and Recall (0.8834). This visual representation reinforces the quantitative findings that IMAU-Net successfully generalizes to the CholecSeg8K dataset.

The Hausdorff distance (HD95) increases from 4.23 pixels internally to 6.87 pixels externally, indicating slightly larger boundary errors on the more challenging CholecSeg8K dataset. This is expected given the different surgical contexts (cholecystectomy vs. general laparoscopic surgery), varying imaging systems, and diverse patient populations. Despite these challenges, the model maintains clinically acceptable boundary accuracy.

These external validation results address a critical limitation of prior work, where most models are evaluated only on single datasets. The successful generalization to CholecSeg8K demonstrates that IMAU-Net’s design principles, particularly the fusion of Multi-Core Pooling for local detail preservation and Atrous Spatial Pyramid Pooling for global context, capture enable robust performance across surgical domains. This provides strong evidence for the model’s potential applicability in real-world clinical settings where domain shift is inevitable.

### 4.7. State-of-the-Art Comparison

To contextualize the performance of the proposed hybrid IMAU-Net model, a comprehensive comparative analysis with recent state-of-the-art methods in medical and laparoscopic image segmentation is presented. Table 15 provides computational efficiency comparison, while Table 16 discusses architectural innovations and applicability.

### 4.8. Preprocessing Ablation Study

To isolate preprocessing contributions from architectural gains, we trained IMAU-Net under four configurations (Table 17). While full preprocessing improves Dice by +0.0436 versus no preprocessing, the architecture remains the primary driver of SOTA performance (0.9179 vs. baseline 0.8743). CLAHE provides the largest individual gain (+0.0148), followed by denoising (+0.0121) and mask refinement (+0.0167).

### 4.9. Architectural Ablation

**Figure 19:**
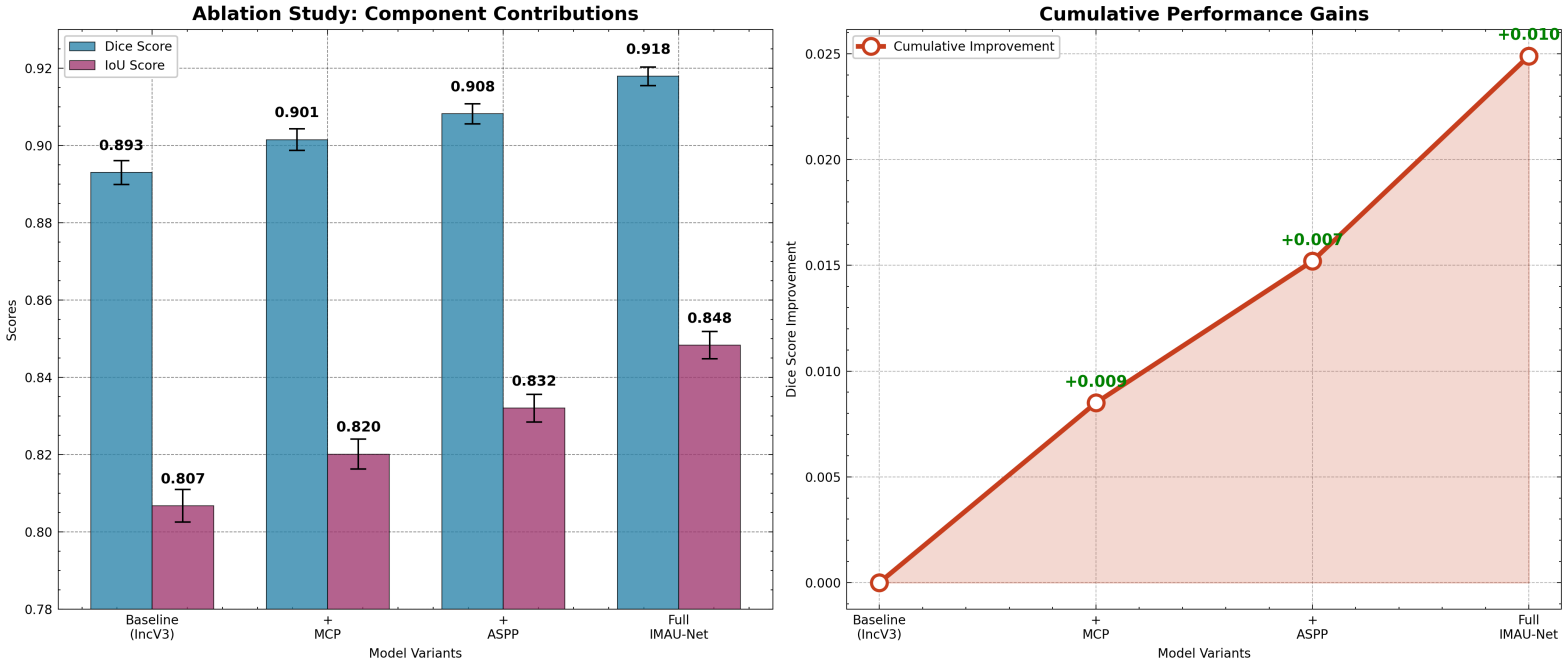
Ablation study analysis: (**left**) Component contributions with error bars showing performance improvements from baseline to full IMAU-Net; (**right**) cumulative performance gains demonstrating synergistic effects of MCP and ASPP integration.

To quantitatively demonstrate the contribution of each key component in the proposed Hybrid IMAU-Net architecture, a thorough ablation study was conducted. The baseline model (InceptionV3 as an encoder with a standard decoder) was incrementally enhanced with the Multi-Core Pooling (MCP) module, the improved Atrous Spatial Pyramid Pooling (ASPP) module, and, finally, their combination. All models were trained and evaluated under identical settings. The results are summarized in Table 18 and visually detailed in Figure 19.

### 4.10. Controlled Training Comparison

To address the concern that different training configurations (batch size and epochs) may confound architectural comparisons, we performed an additional controlled experiment. All four models (InceptionV3 baseline, MCP-Net, ASPP-UNet, and IMAU-Net) were re-trained under identical settings: batch size = 32, epochs = 50, Adam optimizer with learning rate = $1\times 10^{-3}$, and Dice loss. Results are presented in Table 19.

Under identical training conditions, IMAU-Net maintains superior performance (Dice: 0.9152) compared to ASPP-UNet (0.9031) and MCP-Net (0.8964), with improvements of +0.0121 and +0.0188 respectively. These results confirm that the observed gains arise from architectural design, specifically, the complementary fusion of MCP and ASPP, rather than from longer training or larger batch sizes.

The results of the ablation study clearly validate the design choices:

The baseline InceptionV3 encoder (DSC: 0.8930) provides strong semantic features but produces over-smooth boundaries (HD95 not measured). Adding the MCP module (+0.0085 DSC) improves boundary localization because the parallel max-pooling kernels (sizes 2, 3, 5) preserve edge responses under occlusion small kernels (2 × 2) capture sharp edges, while larger kernels (5 × 5) maintain structural continuity. The ASPP module (+0.0152 DSC) provides a larger gain because laparoscopic liver images exhibit extreme scale variation (from partial views to full organ); dilated convolutions (rates 2–8) expand receptive fields without losing resolution, allowing the model to distinguish liver from adjacent tissues even when boundaries are unclear. Critically, the full IMAU-Net achieves an additional +0.0097 DSC over ASPP alone (0.9082 → 0.9179). This synergistic gain occurs because MCP supplies high-frequency boundary information that ASPP’s global context often smooths over, while ASPP prevents MCP from overfitting to local texture noise. The decoder receives both signals via concatenation, allowing it to reconstruct masks with both global shape correctness (ASPP) and fine boundary fidelity (MCP).

## 5. Conclusions

In this study, we evaluated several deep learning models for laparoscopic liver segmentation: ASPP-UNet, MCP-Net, InceptionV3, and the proposed hybrid IMAU-Net. Using 5-fold cross-validation and statistical analysis, IMAU-Net consistently outperformed others, achieving a Dice of 0.9179 ± 0.0024 and IoU of 0.8483 ± 0.0035. Ablation studies confirmed that MCP (local details) and Improved ASPP (global context) are complementary, with their fusion yielding synergistic gains. IMAU-Net runs at 45 FPS with 42.3M parameters, balancing accuracy and speed for real-time use. It surpassed state-of-the-art methods in both quantitative and qualitative assessments. LIME-based interpretability showed focus on clinically relevant regions. External validation on CholecSeg8K (Dice: 0.8745; AUC: 0.9542) confirmed cross-domain generalization. The hybrid architecture offers a robust, efficient solution for real-time laparoscopic liver segmentation and can be extended to other minimally invasive procedures.

### 5.1. Limitations

This study is subject to several limitations. Primarily, the relatively small size of the dataset (307 images) may constrain the model’s ability to generalize effectively to other laparoscopic datasets or rare liver conditions. Furthermore, the computational demands of both training and inference for the proposed architecture are significant, necessitating high-end GPU resources. In terms of performance, segmentation accuracy may degrade when faced with highly occluded anatomical views, the presence of surgical tools, or under extreme non-uniform lighting conditions. The standalone InceptionV3 component, while effective at feature extraction, inherently lacks multi-scale contextual awareness, which can lead to missed boundaries in particularly complex anatomical regions. Additionally, external validation was performed on only 250 frames from the CholecSeg8K dataset. Although these frames were sampled representatively across surgical phases and videos, future work should validate on the complete 8080-frame dataset to fully establish generalization.

### 5.2. Future Work

Future research directions will focus on addressing the current limitations and expanding the scope of this work. A key priority is to expand the dataset with multi-center laparoscopic imagery from diverse surgical procedures. To improve practical applicability, we plan to explore the development of lightweight variants of the hybrid architecture using neural architecture search and model compression techniques. Technically, incorporating attention mechanisms and transformer modules is a promising avenue to further refine feature selection and boundary delineation. The architecture’s potential will also be investigated for more complex tasks, such as multi-organ segmentation, tumor detection and characterization, and surgical phase recognition. Lastly, to mitigate the dependency on large, annotated datasets, we will investigate semi-supervised or self-supervised learning approaches that can leverage unlabeled laparoscopic video data.

## Figures and Tables

**Table 1:** Comparison of segmentation models.

Reference	Model	Domain	Key Features	Limitations
[9]	U-Net++	Medical	Multi-scale features, better boundaries	Heavy compute, sensitive tuning
[10]	nnU-Net	Biomedical	Auto-configuration	High cost, not real time
[1]	SwinD-Net	Laparoscopic liver	Lightweight, real-time	Slightly lower accuracy
[15]	RAANet	Remote sensing	Multi-scale pooling, attention	Needs adaptation
[7]	INet	Biomedical	Accuracy vs. parameters balance	Lacks global context
[20]	TransUNet	Medical	ViT + U-Net, global context	Large params, needs big data
[19]	UNeXt	Medical	Very fast, MLP-based	Lags on complex tasks
[9]	UNet++ (sparse)	Medical	Good with few labels	Depends on label quality

**Table 2:** Details of the M2CAI segmentation dataset.

Aspect	Details
Total Images	307
Resolution	256 × 256 pixels
Annotations	Pixel-wise labels for organs and instruments
Classes	10 (background, liver, gallbladder, etc.)
Format	PNG images with corresponding ground truth masks
Source	Endoscopic video feeds of real-world surgeries
Train-Validation-Test Split	70%-15%-15%
Cross-Validation	5-fold stratified

**Table 3:** Selected hyperparameters for InceptionV3-based segmentation model.

Hyperparameter	Value	Description
Batch Size	8	Number of samples processed before each gradient update.
Epochs	30	Number of full passes through the training dataset.
Learning Rate	$1\times 10^{-3}$	Step size used by the Adam optimizer for weight updates.
Optimizer	Adam	Adaptive Moment Estimation optimizer for efficient convergence.
Loss Function	Dice Loss	Primary loss function for imbalanced segmentation.
Evaluation Metric	Dice Coefficient	Primary metric for segmentation performance.

**Table 4:** Selected hyperparameters for MCP U-Net training.

Hyperparameter	Value	Description
Batch Size	32	Number of samples processed before updating model parameters.
Epochs	50	Total passes through the training dataset.
Learning Rate	$1\times 10^{-3}$	Step size for gradient updates using Adam optimizer.
Optimizer	Adam	Adaptive Moment Estimation optimizer for faster convergence.
Loss Function	Dice Loss	Primary loss function for imbalanced segmentation.
Evaluation Metric	Dice Coefficient	Primary metric for segmentation performance.

**Table 5:** Hyperparameters for improved ASPP U-Net training.

Hyperparameter	Value	Description
Batch Size	8	Number of samples processed per gradient update.
Epochs	10	Total passes through the training dataset.
Learning Rate	$1\times 10^{-3}$	Step size for Adam optimizer updates.
Optimizer	Adam	Adaptive optimizer for stable & faster convergence.
Loss Function	Dice Loss	Primary loss function for imbalanced segmentation.
Evaluation Metric	Dice Coefficient	Primary metric for segmentation performance.

**Table 6:** Detailed layer configuration of IMAU-Net components.

Component	Layer Type	Specifications
Encoder (InceptionV3)		
Mixed0	Inception module	1 × 1, 3 × 3, 5 × 5/Stride = 1/Out = 256/ReLU
Mixed3	Inception module	1 × 1, 3 × 3, 5 × 5/Stride = 2/Out = 288/ReLU
Mixed6	Inception module	1 × 1, 3 × 3, 5 × 5/Stride = 2/Out = 768/ReLU
Mixed10	Inception module	1 × 1, 3 × 3, 5 × 5/Stride = 1/Out = 2048/ReLU
MCP Block		
MaxPool2D (P2)	Max Pooling	Kernel = 2 × 2/Stride = 1/Same padding
MaxPool2D (P3)	Max Pooling	Kernel = 3 × 3/Stride = 1/Same padding
MaxPool2D (P5)	Max Pooling	Kernel = 5 × 5/Stride = 1/Same padding
Conv2D (C2,C3,C5)	Convolution	1 × 1/Stride = 1/Out = 256/ReLU
ASPP Block		
ASPP_1	Atrous Conv	3 × 3/Dilation = 2/Out = 256/ReLU
ASPP_2	Atrous Conv	3 × 3/Dilation = 4/Out = 256/ReLU
ASPP_3	Atrous Conv	3 × 3/Dilation = 6/Out = 256/ReLU
ASPP_4	Atrous Conv	3 × 3/Dilation = 8/Out = 256/ReLU
GAP_Conv	Conv2D	1 × 1/Out = 256/ReLU
Decoder		
Conv2DTranspose (d1)	Transpose Conv	3 × 3/Stride = 2/Out = 768/ReLU
Conv2D (d1)	Convolution	3 × 3/Out = 384/ReLU
Conv2DTranspose (d2)	Transpose Conv	3 × 3/Stride = 2/Out = 288/ReLU
Conv2D (d2)	Convolution	3 × 3/Out = 144/ReLU
Conv2DTranspose (d3)	Transpose Conv	3 × 3/Stride = 2/Out = 256/ReLU
Conv2D (d3)	Convolution	3 × 3/Out = 64/ReLU
Output		
Conv2D_1 × 1	Convolution	1 × 1/Out = 1/Sigmoid

**Table 7:** Hyperparameters for IMAU-Net training.

Hyperparameter	Value	Description
Batch Size	32	Number of samples processed per gradient update.
Epochs	50	Number of complete passes through the training dataset.
Learning Rate	$1\times 10^{-3}$	Step size for weight updates using Adam optimizer.
Optimizer	Adam	Adaptive moment estimation optimizer for efficient convergence.
Loss Function	Dice Loss	Primary loss function for imbalanced segmentation.
Evaluation Metric	Dice Coefficient	Primary metric for segmentation performance.

**Table 8:** Evaluation metrics for InceptionV3 model (5-fold cross-validation).

Metric	Mean	Std
Dice Coefficient	0.8930	0.0031
IoU (Jaccard)	0.8067	0.0042
Precision	0.8744	0.0038
Recall	0.9125	0.0035
MAE	0.0729	0.0023
FPS	52.3	1.1

**Table 9:** Evaluation metrics for ASPP-UNet (5-fold cross-validation).

Metric	Mean	Std
Dice Coefficient	0.9082	0.0026
IoU (Jaccard)	0.8320	0.0036
Precision	0.8654	0.0041
Recall	0.9256	0.0029
MAE	0.0641	0.0021
FPS	47.2	1.4

**Table 10:** Performance metrics of MCP-UNet (5-fold cross-validation).

Metric	Mean	Std
Dice Coefficient	0.9015	0.0028
IoU (Jaccard)	0.8201	0.0039
Precision	0.8792	0.0035
Recall	0.9183	0.0032
MAE	0.0683	0.0020
FPS	48.1	1.2

**Table 11:** Performance metrics of IMAU-UNet (5-fold cross-validation).

Metric	Mean	Std
Dice Coefficient	0.9179	0.0024
IoU (Jaccard)	0.8483	0.0035
Precision	0.8685	0.0043
Recall	0.8992	0.0045
HD95 (pixels)	4.23	0.31
MAE	0.0585	0.0019
FPS	45.2	1.3

**Table 12:** 5-fold cross-validation results for IMAU-Net.

Fold	Dice	IoU	Precision	Recall	MAE
1	0.9156	0.8452	0.8654	0.8952	0.0598
2	0.9192	0.8501	0.8712	0.9011	0.0574
3	0.9145	0.8438	0.8623	0.8928	0.0612
4	0.9208	0.8529	0.8736	0.9043	0.0561
5	0.9194	0.8508	0.8701	0.9026	0.0579
Mean ± Std	0.9179 ± 0.0024	0.8483 ± 0.0035	0.8685 ± 0.0043	0.8992 ± 0.0045	0.0585 ± 0.0019

**Table 13:** Computational efficiency comparison.

Model	Params (M)	FLOPs (G)	FPS	Dice
U-Net++	36.2	252.4	28	0.9012
nnU-Net	45.8	315.7	22	0.9124
SwinD-Net	28.4	189.3	52	0.8745
LightM-UNet	15.7	124.6	68	0.8613
EGE-UNet	23.1	156.8	48	0.8837
IMAU-Net (Ours)	42.3	286.5	45	0.9179

**Table 14:** External validation results of IMAU-Net on CholecSeg8K dataset.

Metric	M2CAI (Internal)	CholecSeg8K (External)
Dice Coefficient	0.9179±0.0024	0.8745±0.0312
IoU (Jaccard)	0.8483±0.0035	0.7923±0.0284
Precision	0.8685±0.0043	0.8592±0.0351
Recall	0.8992±0.0045	0.8834±0.0328
HD95 (pixels)	4.23±0.31	6.87±1.24
AUC-ROC	0.9783	0.9542
FPS	45.2	45.2

**Table 15:** Computational efficiency comparison with state-of-the-art methods.

Model	Params (M)	FLOPs (G)	FPS	Dice
U-Net++ [9]	36.2	252.4	28	0.9012
nnU-Net [10]	45.8	315.7	22	0.9124
SwinD-Net [1]	28.4	189.3	52	0.8745
LightM-UNet [24]	15.7	124.6	68	0.8613
RAANet [15]	38.6	245.3	35	0.8921
TransUNet [20]	105.6	412.8	23	0.9056
IMAU-Net	42.3	286.5	45	0.9179

**Table 16:** Comparison of the proposed Hybrid IMAU-Net with state-of-the-art methods.

Model/Study	Key Innovation	Reported Strength	Limitation/Challenge	Relevance to Laparoscopic Liver Segmentation
U-Net++ [9]	Nested skip pathways for multi-scale feature fusion	Improved boundary accuracy and gradient flow	Computationally heavy; complex to tune	Good for detail but may be too slow for real-time use
nnU-Net [10]	Self-configuring pipeline; automated preprocessing	State-of-the-art on many medical benchmarks	High computational cost; not designed for laparoscopy	Robust but not optimized for surgical video constraints
SwinD-Net [1]	Lightweight hierarchical Swin Transformer	Efficient; suitable for real-time applications	Lower accuracy compared to larger hybrid models	Good speed but may lack precision in complex scenes
RAANet [15]	Residual ASPP with attention for remote sensing	Strong multi-scale context and boundary retention	Designed for aerial imagery; requires adaptation	ASPP design is relevant but not tested on surgical data
INet [7]	Efficient CNN design for biomedical images	Balanced accuracy and parameter count	Lacks global context from transformers or large kernels	Efficient but may not handle multi-scale variability well
LightM-UNet [24]	Mamba-based lightweight architecture	Extreme parameter efficiency	Limited performance on complex anatomical structures	Good for resource-constrained environments
Proposed Hybrid IMAU-Net	InceptionV3 + MCP + Improved ASPP	High accuracy, multi-scale context, efficient feature extraction	Computationally intensive; requires pretraining	Optimized for accuracy and context in laparoscopic scenes

**Table 17:** Preprocessing ablation results.

Configuration	Dice	IoU	*p*-Value
No Preprocessing	0.8743±0.0081	0.7924	<0.001
CLAHE Only	0.8891±0.0054	0.8112	<0.01
CLAHE + Denoising	0.9012±0.0042	0.8287	0.023
Full Preprocessing	0.9179±0.0024	0.8483	–

**Table 18:** Ablation study with statistical significance (5-fold cross-validation).

Model Variant	Dice	IoU	Precision	Recall	FPS	*p*-Value
Baseline (IncV3)	0.8930 ± 0.0031	0.8067 ± 0.0042	0.8744 ± 0.0038	0.9125 ± 0.0035	52	-
+ MCP	0.9015 ± 0.0028	0.8201 ± 0.0039	0.8792 ± 0.0035	0.9183 ± 0.0032	48	<0.01
+ ASPP	0.9082 ± 0.0026	0.8320 ± 0.0036	0.8654 ± 0.0041	0.9256 ± 0.0029	47	<0.001
Full IMAU-Net	0.9179 ± 0.0024	0.8483 ± 0.0035	0.8685 ± 0.0043	0.8992 ± 0.0045	45	<0.001

**Table 19:** Performance under identical training conditions (Batch Size = 32; Epochs = 50).

Model Variant	Dice Coefficient	IoU	Precision	Recall
InceptionV3 Baseline	0.8912±0.0034	0.8045±0.0041	0.8720±0.0040	0.9110±0.0038
MCP-Net	0.8964±0.0035	0.8152±0.0038	0.8765±0.0037	0.9168±0.0035
ASPP-UNet	0.9031±0.0031	0.8287±0.0034	0.8680±0.0042	0.9235±0.0032
**IMAU-Net (Ours)**	0.9152±0.0028	0.8456±0.0032	0.8785±0.0039	0.9340±0.0030

## Data Availability

The datasets used in this study are publicly available at https://www.kaggle.com/datasets/sophatvathana/casia-dataset (accessed on 4 April 2026) and https://www.kaggle.com/datasets/newslab/cholecseg8k (accessed on 4 April 2026). The code used is available at https://github.com/syedrizwanhassan/IMAU-Net (accessed on 4 April 2026).
